# A pragmatic nomogram using routinely collected clinical variables to screen prevalent HFpEF: development and temporal validation

**DOI:** 10.1186/s12872-026-05619-w

**Published:** 2026-02-24

**Authors:** Chunmei Chen, Yuetong Liu, Xinxin Mao, Pengfei Liu, Shuqing Shi, Qingqiao Song, Bingxuan Zhang

**Affiliations:** 1 Department of General Internal Medicine, Guang ’anmen Hospital, China Academy of Chinese Medical Sciences, Beijing, China; 2 Beijing University of Traditional Chinese Medicine, Beijing, China

**Keywords:** Heart failure with preserved ejection fraction, Nomograms, Screening prediction, Chinese population, Logistic models

## Abstract

**Objectives:**

To develop and temporally validate a pragmatic nomogram based on routinely available clinical and laboratory variables to estimate the individualized probability of prevalent heart failure with preserved ejection fraction (HFpEF) at the index assessment, and to support screening or triage, and prioritization for confirmatory echocardiography in settings where comprehensive imaging resources are limited.

**Methods:**

A total of 2187 cases were collected for the prediction model. Another 2026 cases from a new data set were utilized for performing independent temporal validation. The LASSO regression analysis was used to control possible variables. A final screening or triage nomogram for HFpEF was established based on logistic regression, and the discrimination and calibration of the established nomogram were evaluated by bootstrapping with 1000 resamples.

**Results:**

The final nomogram for screening or triage prevalent HFpEF was constructed using nine predictors retained in the final model: Age, systolic blood pressure (SBP), monocyte ratio (MONO%), red cell distribution width–coefficient of variation (RDW-CV), fasting glucose (GLU), triglycerides (TG), high-density lipoprotein cholesterol (HDL-C), Urea, and immunoglobulin G (IgG). The model demonstrated good discrimination, with a C-index of 0.762 in the training cohort and 0.783 in the validation cohort. Calibration plots showed good agreement between predicted and observed probabilities in both cohorts. Internal and temporal validation indicated that the model was robust and reliable.

**Conclusions:**

This cross-sectional screening prediction nomogram estimates individualized risk of prevalent HFpEF using readily non-imaging variables and may support screening or triage and efficient allocation of echocardiography resources across hospital and community workflows. The tool is intended to prioritize referral for echocardiographic confirmation rather than replace guideline-based diagnosis. Prospective multicenter studies are warranted to evaluate transportability, clinical utility, and implementation impact.

**Supplementary Information:**

The online version contains supplementary material available at 10.1186/s12872-026-05619-w.

## Introduction

Heart failure with preserved ejection fraction (HFpEF) is a more heterogeneous syndrome than heart failure with reduced ejection fraction, characterized by a high prevalence of comorbidities, and is more common among the elderly, obese individuals, and women [1]. It accounts for at least half of all diagnosed heart failure cases worldwide, and this proportion is expected to increase with the aging population [2]. The risk factors for HFpEF include obesity, hypertension, and diabetes mellitus, all of which are on the rise, thus contributing to the anticipated increase in HFpEF prevalence. Many patients with HFpEF have coexisting medical conditions, such as pulmonary and renal diseases, which contribute to higher morbidity and mortality [3, 4]. The diagnosis of HFpEF is complex, with significant heterogeneity in clinical practice, resulting in variable clinical courses and prognoses [5]. Even among different guidelines and consensus documents, there are many inconsistencies in the proposed approaches. The lack of a clear definition has undoubtedly hindered the early and consistent diagnosis of HFpEF, which in turn complicates therapeutic research and clinical trials [6]. To date, most pharmacological interventions in HFpEF have yielded neutral results, with no consistent evidence of a definitive mortality benefit, underscoring the ongoing unmet need for effective, phenotype-targeted treatments [4].

Currently, the diagnosis of HFpEF is primarily based on two scoring systems, namely the HFA-PEFF [7] and H_2_FPEF scores [8]. This diagnosis typically requires repeated assessments by specialists to confirm the presence or absence of the disease, which hampers the early identification of HFpEF. Furthermore, the lack of early screening prediction models incorporating common clinical indicators and other factors poses challenges for individuals in effectively managing HFpEF.

From a public health and health-services perspective, delayed recognition of HFpEF can increase downstream morbidity and healthcare utilization, while access to echocardiography remains uneven across care settings. A scalable screening or triage approach using routinely collected clinical information could help identify individuals with a higher likelihood of HFpEF and prioritize confirmatory echocardiography, thereby improving referral efficiency and resource allocation. Therefore, we aimed to develop and validate a pragmatic nomogram using readily available non-imaging variables to estimate the probability of prevalent HFpEF at the index assessment in an electronic health record cohort.

A nomogram, as an intuitive and efficient statistical prediction tool, aids clinicians in comprehensively assessing the probability of HFpEF by considering individual patient characteristics and disease factors. Developing a nomogram that incorporates common clinical indicators is crucial for the early identification of high probability HFpEF populations in China. This tool not only facilitates a collaborative management model involving society, families, and healthcare institutions but also provides a more holistic approach to prevalent HFpEF at the index assessment by thoroughly evaluating the patient’s personal conditions. Furthermore, it can guide the formulation of intervention strategies to some extent. A multi-stakeholder management system helps offer more precise and personalized support, improving health management for individuals at high risk of HFpEF.

## Methods

### Study population

The study is a single-center, cross-sectional prediction study based on electronic health record (EHR) data from the Department of General Medicine at Guang’anmen Hospital, China Academy of Chinese Medical Sciences (Beijing, China). The EHR database includes electronic medical records of adult patients from 2022 to 2024, collected by the hospital’s information center. A separate retrospective EHR dataset comprising adult patients from 2018 to 2021 was used as an independent temporal validation cohort. Both cohorts comprised consecutive adult patients who presented for care for any reason and underwent echocardiography as part of routine clinical assessment, rather than being restricted to a cardiology-only population.

### Outcome definition

**Fig. 1:**
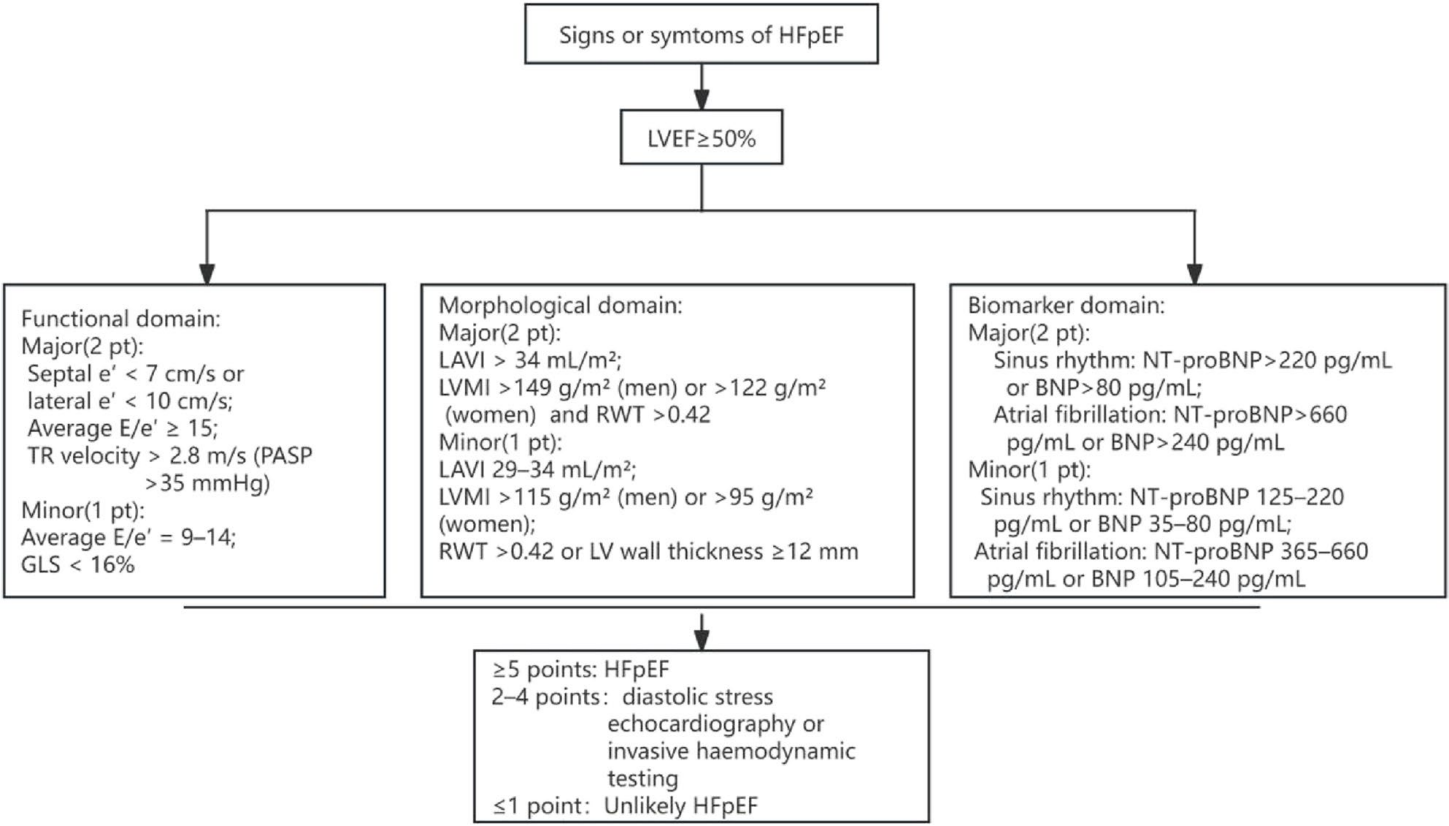
Diagnostic flowchart of HFpEF

The primary outcome was prevalent HFpEF at baseline. HFpEF was diagnosed according to the Heart Failure Association (HFA) of the European Society of Cardiology HFA-PEFF diagnostic algorithm. In brief, HFpEF required: (1) symptoms and/or signs of heart failure; (2) preserved left ventricular ejection fraction (LVEF ≥ 50%) on transthoracic echocardiography; and (3) objective evidence of structural and/or functional cardiac abnormalities and elevated natriuretic peptides. Echocardiographic indicators and NT-proBNP were used to establish the reference-standard diagnosis of HFpEF, but were not included as predictors in the final nomogram. Patients who did not meet HFpEF criteria were classified as the non-HFpEF group, as shown in Fig. 1, Table S1.

This study has been approved by the Ethics Committee of Guang’anmen Hospital. As it utilizes de-identified electronic medical records and does not involve direct human participation, the Ethics Committee of Guang’anmen Hospital, China Academy of Chinese Medical Sciences, has waived the requirement for informed consent.

### Data collection

Clinical data were extracted from the hospital EHR system for both the training and validation cohorts. Each cohort included over 2,000 participants, providing an adequate sample size for model development and temporal validation. At baseline We initially collected 157 candidate predictors and grouped them into five domains: (1) demographics, anthropometrics and vital signs; (2) complete blood count-derived indices and inflammatory markers; (3) serum biochemistry, including cardiac injury markers, lipid and glucose metabolism, liver and renal function, electrolytes, and immunological indices; (4) additional laboratory tests, including coagulation profiles, arterial blood gas parameters, thyroid function tests, and urinary biomarkers; and (5) disease-related diagnostic variables, including NT-proBNP, atrial fibrillation status, and echocardiographic measures. The complete list of candidate predictors is provided in Table S2.

### Predictive variable screening and model establishment and validation

Candidate predictors were selected using a prespecified, clinically oriented pipeline to balance parsimony, interpretability, and practical implementation in routine inpatient care. First, univariable logistic regression was used as an initial dimension-reduction step (*P* < 0.05) to reduce noise from a large candidate set and stabilize subsequent modeling. Second, multicollinearity was assessed using variance inflation factors (VIF). When collinearity was present (VIF > 10), redundant variables were removed based on clinical interpretability, measurement availability, and redundancy within the same physiologic domain to retain a clinically actionable set of predictors. Third, LASSO logistic regression was applied to the reduced predictor set for further regularization and selection, with the penalty parameter (λ) determined by 10-fold cross-validation. multivariable logistic regression model, from which a nomogram was constructed to estimate the probability of prevalent HFpEF for screening or triage.

A multivariable logistic regression model was subsequently fitted using the selected predictors, and a nomogram was developed to estimate the individualized probability of prevalent HFpEF. Model discrimination was assessed using the area under the receiver operating characteristic curve (AUC) and the concordance index (C-index), and calibration evaluated with calibration plots. Decision curve analysis was conducted to quantify clinical net benefit across a range of threshold probabilities. Internal validation was performed using bootstrapping with 1,000 resamples, and independent temporal validation was performed in an independent cohort. The resulting nomogram was intended for clinical application, enabling clinicians to calculate individualized risk probabilities of HFpEF.

Sensitivity analyses were performed using a direct LASSO approach without univariable and VIF pre-screening and bootstrap selection frequency to quantify predictor stability, finally leave-one-predictor-out refitting, to evaluate robustness of predictor selection and model performance.

### Statistical analysis

Statistical analysis was performed using R (version 4.1.3). Baseline characteristics were compared using Student’s *t* tests or the Mann–Whitney *U* tests for continuous variables, as appropriate, and the χ² test for categorical variables. All statistical tests were two-sided, and a *P* value < 0.05 was considered statistically significant. To mitigate overfitting and eliminate non-influential covariates, candidate predictors were first screened using univariate logistic regression, followed by regularized regression for further feature selection. Logistic LASSO regression was implemented with the “glmnet” package, and the optimal penalty parameter (λ) was determined using 10-fold cross-validation. Variables with non-zero coefficients were retained. A prediction nomogram for estimating HFpEF susceptibility was developed using the “rms” package. Discrimination was evaluated by ROC analysis using the “pROC” package, with the area under the AUC quantifying predictive performance. Calibration was assessed to compare predicted and observed probabilities. Clinical utility was evaluated using with the “DCA” package (version 2.0) to estimate net benefit across a range of threshold probabilities.

## Results

### Missing data imputation

In this study, varying degrees of missingness were observed across the candidate variables. Given the relatively large number of variables and the possibility that complex missing data patterns could lead to underestimated uncertainty when using a single imputation approach, we employed multiple imputation by chained equations (MICE) to better capture imputation uncertainty and minimize potential bias. Specifically, we constructed an imputation model incorporating all candidate variables, generated 20 imputed datasets with 10 iterations per dataset, and analyzed each dataset using the prespecified modeling procedure. Parameter estimates, including coefficients, standard errors, and 95%CI, were then combined using Rubin’s rules. Model discrimination was evaluated across imputations and summarized as the mean and the range. For graphical presentation, we used the first imputed dataset as a representative dataset for illustration.

### Screening for predictive factors and nomogram establishment

Initially, univariate logistic regression was applied to 129 candidate variables to screen potential predictors (*P* < 0.05). Significant variables included demographic measures, hemodynamic indices, inflammatory markers, metabolic indice, and renal function markers. The complete list is shown in Table S3. Baseline demographics and clinical characteristics of participants are summarized in Table 1.

**Table 1:** The sub-cohorts demographics and phenotypes of HFpEF

Variables	Total (*n* = 2187)	0 (*n* = 985)	1 (*n* = 1202)	*P*.value
Age	67.00(59.00,77.00)	61.00(52.00,69.00)	72.00(64.00,81.00)	< 0.0001
SBP	134(123,147)	132(120,145)	136(124,149)	< 0.0001
MONO%	5.90(4.90,7.10)	5.60(4.80,6.80)	6.10(5.00,7.30)	< 0.001
RDW-CV	12.80(12.30,13.40)	12.60(12.20,13.20)	12.90(12.40,13.80)	< 0.0001
Glucose	6.14(5.41,8.08)	5.88(5.33,7.55)	6.42(5.53,8.51)	< 0.01
TG	1.31(0.97,1.85)	1.34(0.98,1.90)	1.28(0.97,1.81)	0.04
HDL-C	1.20(1.02,1.43)	1.22(1.06,1.47)	1.19(1.00,1.41)	< 0.001
Urea	5.71(4.63,7.11)	5.35(4.40,6.47)	6.10(4.88,7.99)	< 0.0001
IgG	11.60(9.81,13.43)	11.50(9.70,13.14)	11.68(9.93,13.68)	< 0.01
Gender				0.18
0	1188(54.32)	519(52.69)	669(55.66)	
1	999(45.68)	437(47.31)	533(44.34)	

VIF values were then calculated for these significant variables, ranging from 1.03 to 7496.63. Variables with VIF ≥ 10 were excluded due to multicollinearity, namely MHR, PHR, TCHDLC, TP, ALB, GLB, and CHO. For the least absolute shrinkage and selection operator (LASSO) logistic regression, the λ value ranged from 0.00039 to 0.41900. In our study, a λ value of 0.00039 was selected (Supplementary Fig. 1A and 1B). The elastic net regression model identified 23 variables, from which 9 variables were selected based on clinical and statistical significance: Age, SBP, MONO%, RDW-CV, GLU, TG, IgG, HDL-C and Urea. To quantify model complexity and overfitting risk, we calculated events per variable (EPV) as the number of HFpEF cases in the training cohort divided by the number of predictors retained in the final model.

**Fig. 2:**
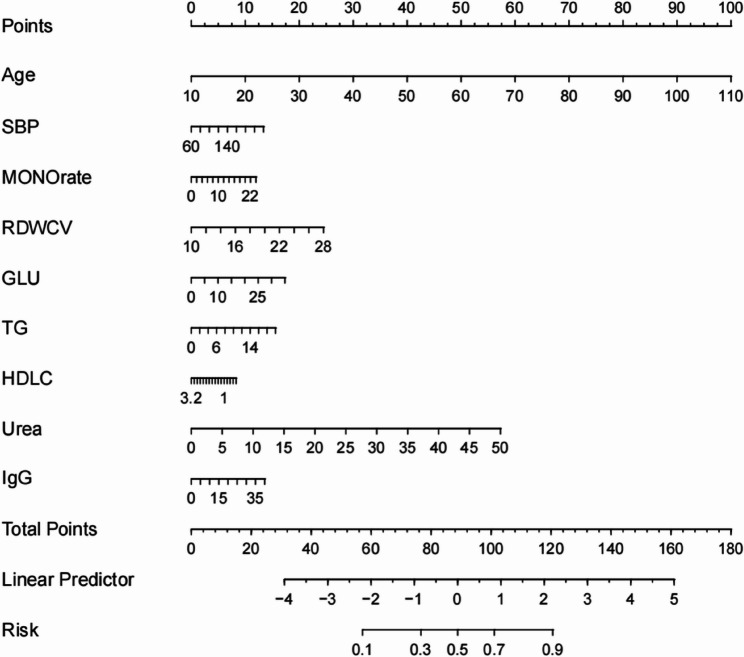
Nomogram for the prediction of HFpEF. Each predictor variable (age, SBP, MONO%, RDW-CV, GLU, TG, HDL-C, Urea, and IgG) corresponds to a specific point value on the upper scales. The total score, obtained by summing the points for all predictors, can be located on the “Total Points” axis, and the corresponding probability of prevalent HFpEF can be read on the “Risk” axis below

To enable individualized prediction of HFpEF probability, a nomogram was constructed based on the results of the multivariate logistic regression model (R² = 0.426) (Fig. 2) [9]. In the nomogram, age emerged as the strongest predictor of HFpEF, followed by Urea, RDW-CV, GLU, TG, IgG, MONO%, and HDL-C. Higher total scores were associated with an increased probability of HFpEF occurrence. The score for each predictor and its corresponding predicted probability of HFpEF are provided in Table S4.

### Performance and net benefit of the model

**Fig. 3:**
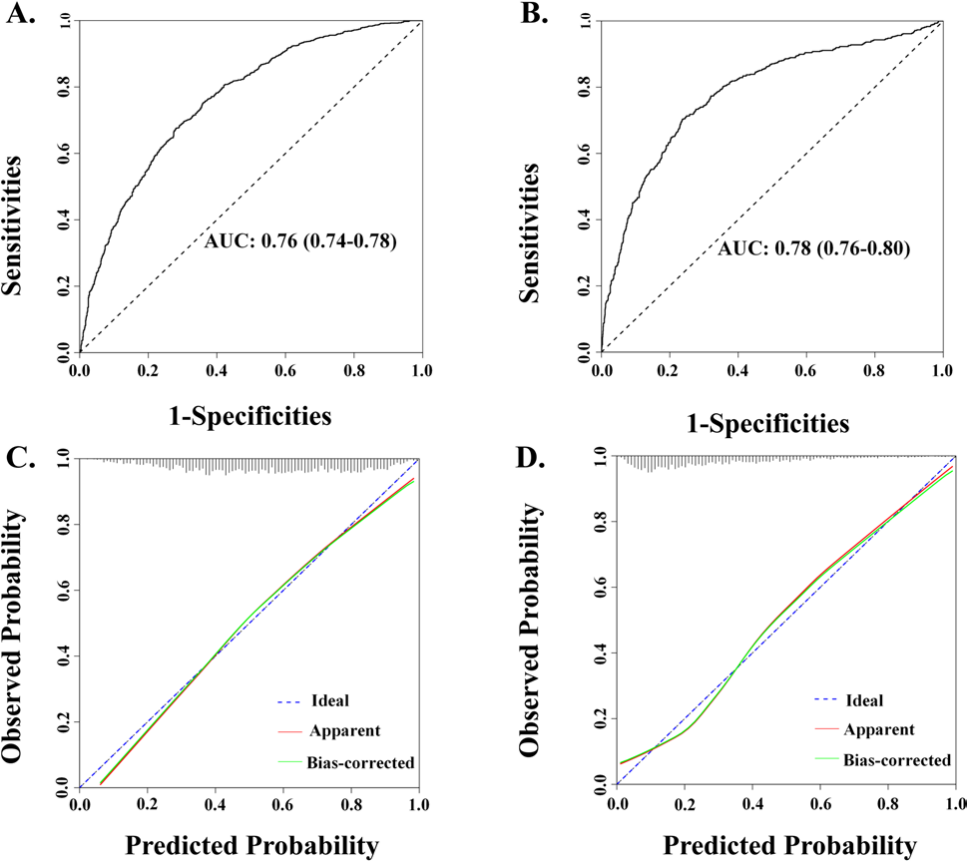
ROC curves in Training (**A**) and Validation cohorts (**B**), and Calibration curves for predicting the probability of HFpEF in Training (**C**) and Validation cohorts (**D**). ROC = receiver operating characteristic; AUC = area under the ROC curve

**Fig. 4:**
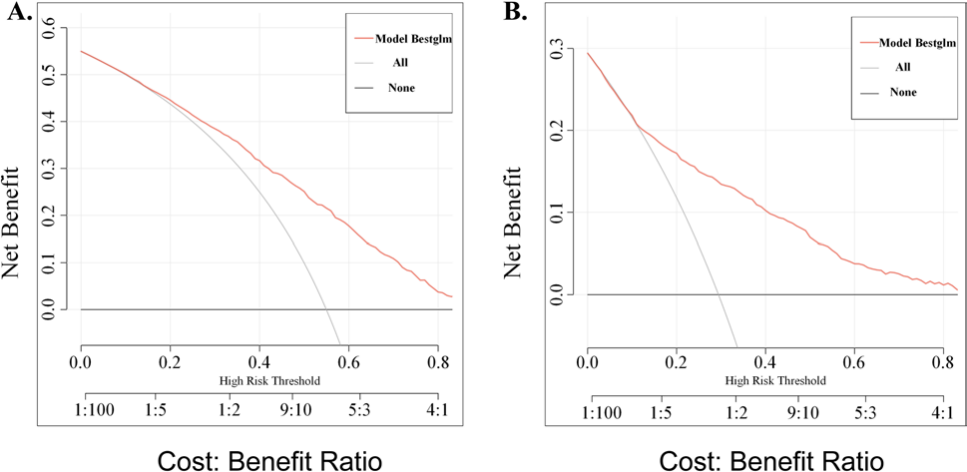
Decision cureve analysis in prediction of HFpEF. **A** Training cohort. **B** Validation cohort

An additional dataset comprising 2,026 cases was included as the validation cohort. In the training cohort, the predictive model demonstrated a C-index of 0.762, while in the validation cohort, the C-index reached 0.783. The area under the ROC curve (AUC) was 0.76 (95% CI: 0.74–0.78) in the training set (Fig. 3A) and 0.78 (95% CI: 0.76–0.81) in the validation set (Fig. 3B). Calibration plots indicated good agreement between predicted and observed probabilities in both datasets (Fig. 3C, D), which showed close agreement between predicted and observed probabilities, suggesting good model reliability. In the validation cohort, intercept was − 1.539 and the calibration slope was 1.058. The Brier score was 0.23 in the validation cohort. Furthermore, decision curve analysis (DCA) demonstrated favorable net clinical benefit in both the training (Fig. 4A) and validation cohorts (Fig. 4B). Decision curve analysis showed that the model yielded a higher net benefit than “test-all” and “test-none” strategies across threshold probabilities ranging from 0.02 to 0.73, supporting its potential utility for screening or triage decisions. Collectively, these findings suggest that the nomogram has promising potential for clinical application.

## Discussion

In this study, we developed and independently validated a cross-sectional prevalent HFpEF screening model to estimate the probability of prevalent HFpEF at the index assessment based on routinely available clinical and laboratory parameters. Using LASSO regression, nine key predictors were selected from over 23 candidate variables, spanning four major domains: (1) age; (2) hematological parameters (RDW-CV and MONO%); (3) renal function-related indicators (Urea and IgG); and (4) metabolic syndrome components (fasting glucose, TG, and HDL-C). The model demonstrated good discrimination (C-index 0.762 in the training set and 0.783 in the validation set), excellent calibration, and favorable clinical net benefit as confirmed by decision curve analysis. These results suggest that our model may facilitate timely identification of individuals with a higher likelihood of prevalent HFpEF and help prioritize confirmatory echocardiography and subsequent management.

### Comparison with existing tools

Current HFpEF clinical algorithms, such as HFA-PEFF and H2FPEF, are primarily designed to support the diagnostic work-up and confirmation of HFpEF and commonly incorporate echocardiographic parameters and/or natriuretic peptides. These requirements may limit feasibility in primary care and community settings where timely access to echocardiography and specialist interpretation is constrained. In contrast, our nomogram uses routinely available demographic, metabolic, inflammatory, and renal indicators obtainable from standard clinical visits or health check-ups, enabling scalable screening or triage and prioritization for confirmatory echocardiography. Notably, we temporally validated the model in an independent dataset, which supports transportability and potential real-world utility across care settings.

In this cross-sectional diagnostic prediction study, the nomogram estimates the individualized probability of prevalent HFpEF at the point of care. Its intended role is complementary to guideline-based assessment: to streamline referral pathways and allocate echocardiography resources to individuals most likely to benefit from further evaluation, rather than to replace imaging-based diagnosis. In practice, clinicians may choose an operating threshold probability according to local echocardiography capacity and the relative harms of false positives versus missed HFpEF. For example, a lower threshold can be used to maximize sensitivity in resource-adequate settings, whereas a higher threshold may prioritize specificity when imaging resources are constrained.

### Clinical implications

#### Ageing and hemodynamic stress

Age is a leading risk factor for HFpEF, with epidemiological studies consistently demonstrating a strong correlation between ageing and HFpEF prevalence [10]. The ageing process itself induces myocardial stiffness and diastolic dysfunction, key hallmarks of HFpEF. Elevated SBP exacerbates ventricular-vascular uncoupling, and chronic hypertension induces concentric remodeling and myocardial fibrosis, promoting elevated LV filling pressures [11, 12].

#### Inflammatory and immune activation

RDW-CV, an inexpensive hematological marker, has been linked to diastolic dysfunction, neurohormonal activation, and adverse prognosis [13–22]. Elevated monocyte ratio reflect systemic inflammation and correlate with increased BNP levels, LV mass index, and left atrial volume, supporting the hypothesis that inflammation drives early myocardial fibrosis. Moreover, IgG participates in Fcγ receptor activation and complement cascade engagement, triggering cardiomyocyte injury and electrophysiological abnormalities [23–26].

#### Renal and neurohormonal axis

Urea elevation reflects not only renal dysfunction but also neurohormonal activation (RAAS, AVP) and systemic inflammation [27–30]. BUN levels correlate with left atrial volume index and are associated with vascular calcification and oxidative stress, which may exacerbate diastolic dysfunction.

#### Metabolic dysregulation

Hyperglycemia and insulin resistance shift myocardial energy metabolism toward fatty acid oxidation, activate the NLRP3 inflammasome, and promote myocardial inflammation and fibrosis [31–36]. Elevated TG and TyG index predict all-cause mortality and rehospitalization, likely via lipotoxic effects, endothelial dysfunction, and microvascular rarefaction [37–41]. Low HDL-C further aggravates endothelial dysfunction and loss of microvascular integrity, whereas HDL-C exerts anti-inflammatory and anti-fibrotic effects [42].

Collectively, these findings emphasize that HFpEF is a multifactorial syndrome involving aging, inflammation, metabolic dysregulation, and cardiorenal interaction—precisely the domains captured by our nomogram. This comprehensive approach may enable early identification of higher likelihood of prevalent HFpEF individuals and guide timely lifestyle modification, blood pressure control, and metabolic optimization.

### Strengths and innovation

Our study has several notable strengths. First, it is based on a large sample size, with over 2,000 participants in both the training and validation cohorts, ensuring adequate statistical power, and it employed LASSO regression for variable selection to minimize overfitting. Second, we conducted temporal validation and decision curve analysis, which enhance the model’s generalizability and demonstrate its net clinical benefit. Third, the integration of novel biomarkers—including inflammatory and immune markers as well as renal indicators—extends risk prediction beyond traditional metabolic and hemodynamic parameters, providing a more comprehensive assessment of HFpEF risk. Finally, the nomogram is simple, visual, and easily implementable in community screening programs and electronic health record systems, underscoring its potential for real-world clinical application.

## Limitations and future directions

This study is subject to several limitations. First, the design is cross-sectional and estimates the probability of prevalent HFpEF at the index assessment; it does not predict incident HFpEF or long-term outcomes, and causal inference is not supported; Second, we used an independent 2018–2021 cohort for temporal validation, both cohorts originated from the same center; therefore, transportability to other regions, healthcare systems, or ethnic groups remains uncertain and true geographic external validation is required; Third, Because HFpEF adjudication required echocardiography and natriuretic peptides, only hospitalized patients who underwent echocardiography could be assigned an outcome label and were eligible for model validation. Patients without echocardiography were not selectively excluded for clinical reasons but were unverifiable for the reference standard, which may introduce verification bias and limit generalizability to unselected outpatient or health-check populations. Model performance may differ in settings with different echocardiography referral thresholds; Fourth, routinely measured laboratory variables may vary across institutions due to assay calibration and measurement practices, which may affect reproducibility. Fifth, key HFpEF-related clinical features were not available in a sufficiently standardized form for inclusion, which may reduce discrimination in borderline or atypical presentations. Finally, the C-index indicates moderate discrimination; prospective implementation studies are needed to quantify real-world net benefit, echocardiography utilization, and downstream clinical impact. Future work should include multicenter geographic validation, refinement with additional clinical variables, and prospective evaluation of workflow integration.

## Conclusion

In this study, we developed and independently validated a pragmatic nomogram to estimate the individualized probability of prevalent HFpEF at the index assessment using nine routinely available clinical variables. The model showed good discrimination, excellent calibration, and favorable clinical net benefit, supporting its potential use for screening or triage and echocardiography prioritization in both hospital and community settings. By integrating demographic, inflammatory, metabolic, and renal indicators, the nomogram captures key domains relevant to HFpEF. Given the cross-sectional design, this tool should be interpreted as a screening or triage model rather than a prognostic model for future HFpEF events or a classifier between HFpEF and HFrEF. Future multicenter prospective studies are warranted to further evaluate transportability and clinical impact.

## Supplementary Information

Supplementary Material 1.

Supplementary Material 2.

Supplementary Material 3.

Supplementary Material 4.

Supplementary Material 5.

Supplementary Material 6.

Supplementary Material 7.

## Data Availability

The datasets generated and analyzed during the current study are not publicly available due to privacy restrictions but are available from the corresponding author on reasonable request.
