# Supplementary material for: A pragmatic nomogram using routinely collected clinical variables to screen prevalent HFpEF: development and temporal validation
Source: BMC Cardiovasc Disord. 2026 Feb 24;26:268. doi: 10.1186/s12872-026-05619-w (PMC13037100; doi:10.1186/s12872-026-05619-w)
Supplement: Supplementary file 6 — Supplementary Material 6. [file 12872_2026_5619_MOESM6_ESM.docx]

**Table S5. Discrimination of the primary 9-variable nomogram and predictor-deletion sensitivity analyses in temporal validation.**

| **Model** | | **Predictors** | **Training AUC**  **(mean)** | **Training**  **AUC**  **(range)** | **Validation**  **AUC**  **(mean)** | **Validation**  **AUC**  **(range)** |
| --- | --- | --- | --- | --- | --- | --- |
| Primary model | Age, SBP, MONO#, RDW-CV,  GLU, TG, HDL-C, Urea, IgG | | 0.762 | 0.759-0.765 | 0.740 | 0.730-0.746 |
| M1 | Age, SBP, MONO#, RDW-CV,  GLU, TG, HDL-C, Urea | | — | — | 0.740 | 0.730-0.746 |
| M2 | Age, SBP, MONO#, RDW-CV,  GLU, HDL-C, Urea | | — | — | 0.738 | 0.728-0.744 |
| M3 | Age, Urea, RDW-CV, SBP, GLU | | — | — | 0.732 | 0.724-0.738 |

Table S5. Across imputations, AUC estimates were stable with narrow ranges. Removing low-selection-frequency predictors yielded nearly identical discrimination, indicating robustness of model performance to reasonable variations in predictor inclusion (multiple imputation, m=20).

**Table S6. Robustness checks for predictor selection strategy**

| **Handle** | **Group** | **AUC** | **95% CI** |
| --- | --- | --- | --- |
| univariate-VIF-LASSO | Train | 0.76 | 0.74-0.78 |
| direct LASSO | Test | 0.77 | 0.75-0.80 |

Table S6. Comparison between the direct LASSO model and the original model. Direct LASSO logistic regression (λ = 1se) without univariate screening

| Predictor | Selection frequency (%) |
| --- | --- |
| Age | 100% |
| Urea | 99% |
| RDW-CV | 62% |
| SBP | 39% |
| GLU | 34% |
| HDL_C | 20% |
| MONO_rate | 17% |
| IgG | 7.5% |
| TG | 5% |

**Table S7. Bootstrap selection frequency within the 9-predictor set (B=200)**

Table S7. Core predictors (Age, Urea, RDW-CV) showed high selection frequencies, while several predictors demonstrated smaller marginal contributions; consistent with this, deletion of IgG and/or TG had negligible impact on discrimination.
