# Supplementary figures and images for: A pragmatic nomogram using routinely collected clinical variables to screen prevalent HFpEF: development and temporal validation
Source: BMC Cardiovasc Disord. 2026 Feb 24;26:268. doi: 10.1186/s12872-026-05619-w (PMC13037100; doi:10.1186/s12872-026-05619-w)

**Figure S1. Annual number of hospitalized patients classified as HFpEF from 2018 to 2024.**


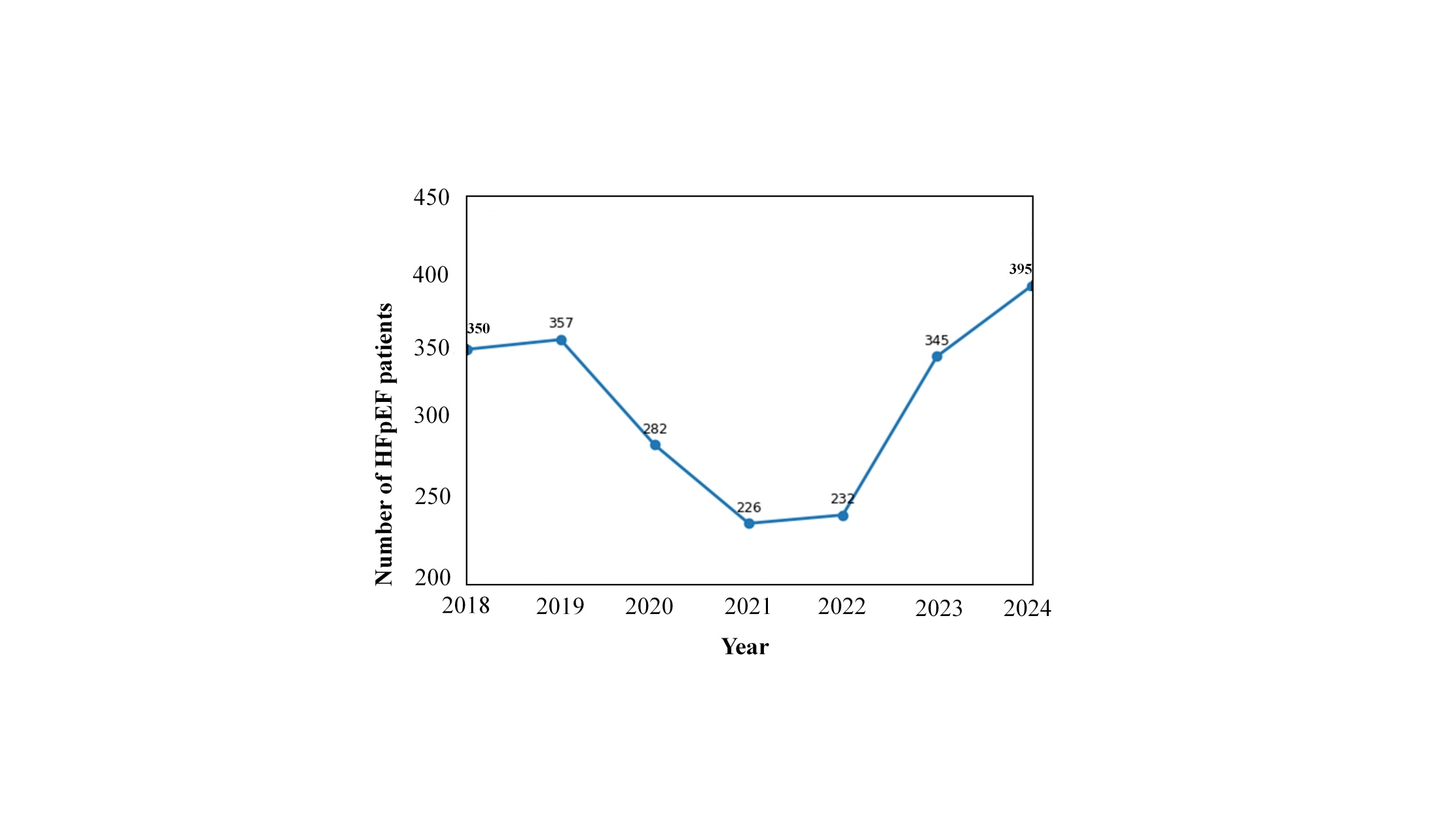

Supplement: Supplementary file 7 — Supplementary Material 7. [file 12872_2026_5619_MOESM7_ESM.docx]
